# Providing surgical care in Somalia: A model of task shifting

**DOI:** 10.1186/1752-1505-5-12

**Published:** 2011-07-15

**Authors:** Kathryn M Chu, Nathan P Ford, Miguel Trelles

**Affiliations:** 1 Médecins sans Frontières, 49 Jorrisen St, Braamfontein 2017, Johannesburg, South Africa; 2 Departments of Surgery and International Health, Johns Hopkins University, Baltimore, MD, USA; 3 Faculty of Health Sciences, Simon Fraser University, Vancouver, Canada; 4 Médecins sans Frontières, rue Dupré 94, 1090 Brussels, Belgium

## Abstract

**Background:**

Somalia is one of the most political unstable countries in the world. Ongoing insecurity has forced an inconsistent medical response by the international community, with little data collection. This paper describes the "remote" model of surgical care by Medecins Sans Frontieres, in Guri-El, Somalia. The challenges of providing the necessary prerequisites for safe surgery are discussed as well as the successes and limitations of task shifting in this resource-limited context.

**Methods:**

In January 2006, MSF opened a project in Guri-El located between Mogadishu and Galcayo. The objectives were to reduce mortality due to complications of pregnancy and childbirth and from violent and non-violent trauma. At the start of the program, expatriate surgeons and anesthesiologists established safe surgical practices and performed surgical procedures. After January 2008, expatriates were evacuated due to insecurity and surgical care has been provided by local Somalian doctors and nurses with periodic supervisory visits from expatriate staff.

**Results:**

Between October 2006 and December 2009, 2086 operations were performed on 1602 patients. The majority (1049, 65%) were male and the median age was 22 (interquartile range, 17-30). 1460 (70%) of interventions were emergent. Trauma accounted for 76% (1585) of all surgical pathology; gunshot wounds accounted for 89% (584) of violent injuries. Operative mortality (0.5% of all surgical interventions) was not higher when Somalian staff provided care compared to when expatriate surgeons and anesthesiologists.

**Conclusions:**

The delivery of surgical care in any conflict-settings is difficult, but in situations where international support is limited, the challenges are more extreme. In this model, task shifting, or the provision of services by less trained cadres, was utilized and peri-operative mortality remained low demonstrating that safe surgical practices can be accomplished even without the presence of fully trained surgeon and anesthesiologists. If security improves in Somalia, on-site training by expatriate surgeons and anesthesiologists will be re-established. Until then, the best way MSF has found to support surgical care in Somalia is continue to support in a "remote" manner.

## Background

Somalia, located in East Africa, is one of the most political unstable countries in the world. The central government collapsed in 1991 when President Siad Barre was ousted during a coup and since then civil war between various clan leaders has led to lawlessness, and insecurity. Currently the country is divided into several parts that are nearly ruled autonomously. In addition to ongoing insecurity, Somalia is plagued by environmental disasters such as drought and flood leading to health emergencies and provoking conflicts over scarce resources. Most social services including health care have collapsed; under- 5 mortality rate is one in four and life expectancy is approximately 50 years [1].

Despite substantial reliance on external humanitarian assistance, ongoing insecurity has limited the ability of international organizations to provide medical care as some risks such as kidnapping are higher for expatriate staff compared to local staff. As a consequence, there has been little data collection and very few reports of humanitarian assistance programmes in Somalia.

Médecins Sans Frontières (MSF) has been providing healthcare in Somalia since the late 1980s. However, in country support has been limited in recent years due to insecurity. In order to continue to provide care in this context, some programs are managed remotely via expatriate teams located in neighboring countries such as Kenya. While limited contact with ground staff means less accountability and oversight, this is the only feasible way to support care in this unstable setting. This paper describes the remote model of surgical care by Medecins Sans Frontieres, in Guri-El, Somalia. The challenges of providing the necessary prerequisites for safe surgery are discussed as well as the successes and limitations of task shifting in this resource-limited context.

## Methods

### Somalia

The MSF healthcare response in Somalia has responded to a diversity of needs, ranging from primary care and tuberculosis control programs to the provision of emergency trauma and obstetrical surgical services. Prior to 2008, local staff were supervised by permanent expatriates, but following the killing of three staff members in Kismayo by a targeted roadside bomb, expatriates were prohibited from working in the country for security reasons. Currently, MSF's projects in Somalia are run by local staff, with material and financial support provided by an international co-ordination team based in Nairobi, Kenya.

### Istarlin Hospital, Gur-El, Galguduud

The Galguduud region is located in central Somalia and has a population of approximately 377,000. In January 2006, MSF opened a project in Guri-El located between Mogadishu and Galcayo. The objectives were to reduce mortality due to complications of pregnancy and childbirth and from violent and non-violent trauma. MSF based itself in a private facility, the 80-bed Istarlin Hospital, which received patients from the surrounding 250 km. The hospital operating room was in disrepair: sterilization was not properly done, and clean water and electricity were not readily available.

At the start of the program, expatriate surgeons and anesthesiologists established safe surgical practices. Specific guidelines concerning disinfection of surgical linen, sterilization of surgical instruments, essential medications, blood transfusions, the organization of the surgical and operating theatre departments, nursing care, and the layout of the health structures were developed. Protocols regarding antibiotic therapy and prophylaxis, post-operative pain management, indications for Cesarean section, anesthesia for pediatrics and obstetrics, and oxygen therapy were implemented. These guidelines and protocols were used to train the local staff to manage the surgical ward, sterilization, and the operating theatre. Technical training in surgical and anesthesia skills were also provided.

In January 2008, MSF's permanent expatriate presence ended due to increased insecurity. Since then, the surgical program has been run remotely from Nairobi, Kenya by a team consisting of a head of mission, a medical coordinator, an administrator, and a project coordinator. Visits are made to Istarlin at least twice a year in order to ensure that MSF standards, protocols, and guidelines are being followed in peri-operative care.

Surgical care is provided by a Somalian doctor with surgical skills who is extremely competent, especially in trauma surgery. He trained under MSF's expatriate surgeons for two years prior to the end of their presence. He also worked with two other non-governmental organizations, the International Committee for the Red Cross and the International Medical Corps, for several years and was mentored by expatriate surgeons. He has attended several training seminars including a WHO surgical training course in Mogadishu. This doctor with surgical skills must function independently. He does not perform elective surgery. Mogadishu has the closest referral hospital but is over 200 km away. MSF does not provide ambulance services due to security constraints, but cases are discussed with the surgeons there. MSF surgeons are also available by email consultation. A surgical nurse who has received informal on-the-job training, also performs procedures, mostly emergency obstetrics and minor operations. All anesthetics are given by anesthetic nurses.

### Data Sources

This review describes surgical interventions done between October 2006 and December 2009; all procedures that required anesthesia and were performed in the operating room were considered as surgical interventions. Data was prospectively collected in an electronic database. Baseline characteristics on age, gender, military status, and American Society of Anesthesiology (ASA) physical status classification as well as data on surgical pathology, procedure type, and operative mortality were recorded in the database at the time of the procedure. Surgical pathology was grouped into the following categories: obstetric emergencies, infection, neoplasm, accidental injury, violence-related injury, and other.

### Statistical analysis

Baseline characteristics were described using medians and interquartile ranges (IQRs) for continuous variables and counts and percentages for categorical data. Logistic regression was used to model associations with violence-related injury. Variables considered in the analysis included age, gender, military status, ASA classification, and blood transfusions. Factors with a p < 0.1 on univariate analysis were included in a multivariate model. All tests and confidence intervals were considered to be significant at a p ≤ 0.05. All analyses were performed using STATA 10 (College Station, TX, USA).

## Results

Between October 2006 and December 2009, 2086 operations were performed on 1602 patients (24% re-interventions). The majority (1049, 65%) were male and the median age was 22 (interquartile range, 17-30), with 152 patients (6%) under 5 years of age. 20% of patients were in the military. 1460 (70%) of interventions were emergent. 1649 (79%) of procedures were performed under general anesthesia without intubation, 300 (14%) under local anesthesia, 55 (3%) under spinal anesthesia, and 40 (2%) under general anesthesia with intubation. There were 8 cases of operative mortality (0.5% of all surgical interventions) among which 4 were trauma- related and 4 were obstetric-related. Hospital mortality was unknown.

### Surgical Pathology

Trauma accounted for 76% (1585) of all surgical pathology: 45% (939) were due to violent-related injury and 31% (652) due to accidental injury. Obstetrical emergencies accounted for 14% (284) of interventions, infection 6% (128), and neoplasms 0.3% (7). Gunshot wounds accounted for 89% (584) of violent injuries (Table 1). The most common non-violence-related injuries were burns and falls. Wound debridement and suturing were the most common procedures for trauma. Only 7% (111) of trauma cases required abdominal surgery and only 5% (73) were orthopedic related. (Table 2).

**Table 1:** Causes of Violent Injury

	N	(%)	
Gunshot Wound	584	(89)	
Knife	55	(8)	
Torture	12	(2)	
Bombs	6	(1)	

Total	657	100	

**Table 2:** Trauma and Non-Trauma Related Interventions

Trauma	N	(%)	Non-Trauma	N	(%)
Wound Debridement	674	(42)	Cesarean section	161	(33)
Suturing	465	(29)	Suturing, I and D, Circumcision	85	(17)
Abdominal Surgery/Bowel Resection	111	(7)	Wound Debridment	55	(11)
Dressing Changes under Sedation	75	(5)	Dressing Changes under Sedation	28	(6)
Fracture Reductions	56	(4)	Abdominal Surgery*	20	(4)
Amputations	17	(1)	Tubal ligation/Dilation and curettage	19	(4)
Skin Grafts	7	(0.5)	Minor Surgery**	12	(2)
Other	143	(9)	Other	39	(8)

Total	1591	(100)	Total	495	(100)

*Bowel resection, appendectomy, tumour resection

**Herniorraphy, hydrocelectomy, hemmorrhoidectomy

### Associations with Violence-related Injury

Male gender (adjusted odds ratio (AOR) = 7.7, P < 0.001), military status (AOR = 2.7, P < 0.001), and age > 15 years (AOR = 3.3 P < 0.001) were associated with violence-related injury (Table 3).

**Table 3:** Associations with Violence-related Injury

	**Univariate**			**Multivariate**		
	
	**OR**	**95% CI**	**P**	**OR**	**95% CI**	**P**
		
						
Female	1.0					
Male	9.9	(7.5-13.2)	< 0.001	7.7	(5.6-10.8)	< 0.001
						
Age < 15 years	1.0					
Age ≥ 15 years	3.8	(2.8-5.2)	< 0.001	3.3	(2.3-4.7)	< 0.001
						
Civillian	1.0					
Military	6.3	(4.7-8.6)	< 0.001	2.7	(1.9-3.7)	< 0.001

### Task shifting

All surgical procedures were performed by non-surgeons (doctor with surgical skills and a surgical nurse) after January 2008. From 2008-2009, the doctor with surgical skills performed 1119 (78%) of procedures and the surgical nurse 314 (22%). The surgical nurse performed 46% (46) of all Cesarean sections and 60% (35) of uterine evacuations. The doctor performed the majority (89%, 306) of elective cases. Peri-operative mortality was lower (0.2%, 2 cases) between 2008-2009 compared to 2006-2007 (1.7%, 6 cases), P < 0.001).

## Conclusions

There are very few published outcome reports from surgical services in war-torn resource-limited settings. In this programme, nearly half of surgical interventions were for violence-related trauma and another third were due to accidental trauma. Most interventions were relatively minor procedures such as wound debridement, suturing, or dressing changes, with only a small number of trauma cases requiring abdominal surgery or advanced orthopedic knowledge. While this may partly reflect the preference of the lesser-trained surgical staff to deal with less complicated cases, the caseload is similar to findings in other African district hospitals [2], and strongly suggests that in resource-limited conflict areas most surgical interventions could be performed by non-surgeons, which is an important consideration given the lack of local surgeons in resource-limited settings [3] and the danger posed to expatriate surgeons.

Somalia has one of the highest maternal mortality ratios in the world (> 1000 deaths per 100,000 live births compared to 9 per 100,000 live births in resource-rich countries) [4] due to poor access to emergency obstetric care. In this program, Cesarean sections represented a lower proportion of surgical interventions compared to reports from other conflict settings [5]. Istarlin Hospital provides the only emergency obstetrical service for the region therefore patients are unlikely to be seeking care elsewhere. Currently, only 50 Cesarean sections are performed annually in the Galgaduud region and the estimated Cesarean rate is < 1%. The WHO recommends that 5-15% of deliveries should be delivered by Cesarean section [6]. A lower proportion suggests that some women in the community with complicated deliveries may not be accessing care. It is estimated that less that 2% of women in Somalia deliver at a health care facility with a skilled attendant [7]. This is likely due to a combination of factors such as lack of facilities, insecurity of road travel, the inequality of women, and the fear of institutional deliveries [8,9]. The reasons for such low uptake of emergency obstetrics requires further investigation.

The most common type of anesthesia provided in this program was general anesthesia without intubation which is safer than general anesthesia with intubation for nurse-anesthetists or anesthesia providers that are informally trained. However, the proportion of cases performed under spinal anesthesia was low and this was likely due to the inexperience of the practitioners. More training is needed to increase the capacity of the anesthetic nurses.

Task shifting is an essential component of this program. For the past three years, surgical services have been provided by non-surgeons (a doctor with surgical skills and a surgical nurse) and anesthesia by non-anesthesiologists (anesthetic nurses). Such task shifting was a consequence of the high insecurity in Somalia, as most surgical programmes run by MSF involve expatriate surgeons and anesthesiologists. However, task shifting is increasingly acknowledged as being an important approach to overcoming specialized human resource shortages more generally: specialist physicians such as surgeons and anesthesiologists are scarce in sub-Saharan Africa [3], and in many settings non-surgeons are responsible for providing the majority of surgical care [10]. The types of procedures performed are limited both by the technology and equipment available as well as the skills of these practitioners. In certain countries, specific surgical procedures such as emergency obstetrical care or orthopedic trauma are safely performed safely performed by non-doctors [11-14]. In low-income settings such as Niger, Malawi, and Mozambique, surgical task-shifting has resulted in an increased provision in essential surgical services [15,16]. For task shifting to be successful, several conditions are required such as regular supervision and exposure to technologic updates. Any practitioner working in isolation can fall into the trap of inadvertently making the same mistakes and developing improper techniques and/or make incorrect decisions. For the Somalian doctor and nurse, options for supervision are limited in county, and it is currently too dangerous for expatriate surgeons to make field visits for any length of time to do meaningful training. MSF is providing them additional training in Kenya. While it is difficult to evaluate the quality of surgical care, this report shows that the peri-operative mortality, a crude measure of the quality of surgical services, was not higher after expatriates left the program (in fact, it decreased). This demonstrates that safe surgery is possible while task shifting and in this resource-limited setting.

The delivery of surgical care in any conflict-settings is difficult, but in situations where international support is limited, the challenges are more extreme. However, in settings that are too insecure to provide permanent on-the ground support, the remote model is a feasible way to deliver emergency surgical services. In our program, logistical and financial support was provided from neighboring (more stable) Kenya. Task shifting, or the provision of services by less trained cadres, was utilized and peri-operative mortality remained low demonstrating that safe surgical practices can be accomplished even without the presence of fully trained surgeon and anesthesiologists. Well-established protocols and guidelines helped maintain the quality of care. The remote model of surgery lacks regular oversight by fully trained surgeons and anesthesiologists, so evaluations and trainings can only be carried out a few times a year. The program could be improved with more training of Somalian staff; discussions are already underway for extra surgical and anesthesia training outside Somalia for the doctor and nurses. Live consultations via videoconferencing for difficult cases would also be beneficial. If security improves in Somalia, permanent expatriate presence will be re-established. Until then, the best way MSF has found to support surgical care in Somalia is continue to support in a "remote" manner.

## Competing interests

The authors declare that they have no competing interests.

## Authors' contributions

KC, PN, NF, and MT were responsible for the overall concept and design. KC, PN, and MT contributed to the data collection and analysis. KC, NF, and MT contributed to intellectual content, and writing of the paper. KC wrote the first draft of the paper. All authors reviewed and approved the final version of the paper.
